# Identification of compounds from chufa (*Eleocharis dulcis*) peels by widely targeted metabolomics

**DOI:** 10.1002/fsn3.3085

**Published:** 2022-10-03

**Authors:** Guanli Li, Shuangquan Huang, Xiaochun Li, Yanghe Luo, Hui Nie

**Affiliations:** ^1^ Guangxi Key Laboratory of Health Care Food Science and Technology Hezhou University Hezhou Guangxi China; ^2^ School of Food Science and Technology Dalian Polytechnic University Dalian Liaoning China

**Keywords:** Chinese water chestnut, metabolite profiles, peels, UPLC‐MS

## Abstract

The Chinese water chestnut (CWC) is among the most widespread and economically important vegetables in Southern China. There are two different types of cultivars for this vegetable, namely, big CWC (BCWC) and small CWC (SCWC). These are used for different purposes based on their metabolic profiles. This study aimed to investigate the metabolite profile of CWC and compare the profiles of peels collected in different harvest years using ultraperformance liquid chromatography/mass spectrometry (UPLC–MS)‐based metabolomics analysis. Three hundred and twenty‐one metabolites were identified, of which 87 flavonoids, 25 phenylpropanoids, and 33 organic acids and derivatives were significantly different in the content of the two varieties of BCWC and SCWC. The metabolite profiles of the two different cultivars were distinguished using principle component analysis (PCA) and orthogonal projections to latent structures discriminant analysis, and the results indicated differences in the metabolite profile of *Eleocharis dulcis* (Burm. f.) Trin. ex Hensch. Three isomers of hydroxycoumarin, namely, *O*‐feruloyl‐4‐hydroxycoumarin, *O*‐feruloyl‐3‐hydroxycoumarin, and *O*‐feruloyl‐2‐hydroxycoumarin, exhibited increased levels in BCWC, while p‐coumaric acid and vanillic acid did not show any significant differences in their content in BCWC and SCWC peels. This study, for the first time, provides novel insights into the differences among metabolite profiles between BCWC and SCWC.

## Graphical abstract

The big Chinese water chestnut (BCWC) and small Chinese water chestnut (SCWC) peels using ultraperformance liquid chromatography/mass spectrometry (UPLC/MS)‐based metabolomics analysis. A total of 321 significant metabolites, including 90 flavonoids, 25 phenylpropanoids, and 33 organic acids and derivatives, were identified/annotated from BCWC and SCWC. In total, flavonoids and phenylpropanoids were identified with different levels, and which higher levels were found in SCWC than in BCWC.

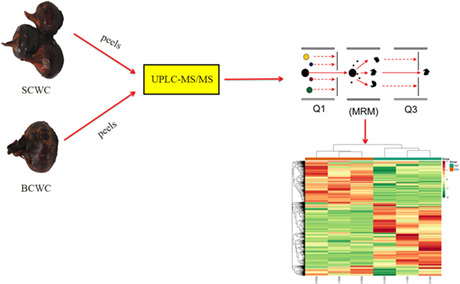

## 1 INTRODUCTION

The Chinese water chestnut (CWC) or chufa (*Eleocharis dulcis* (Burm. f.) Trin. ex Hensch.), which belongs to the family Cyperaceae and whose Chinese name is “Ma Ti (MT)”, is widely found and one of the most economically important vegetables in southern China, particularly in the Guangxi Province (Luo et al., 2014; Nie et al., 2018), most notably in Guilin and Hezhou. Based on the differences in size and usage, the CWC can be categorized into two types: the big size CWC, which is an edible fruit, whose peel can be eaten as fresh food or as a side dish and henceforth is referred to as big CWC (BCWC), and the small CWC, from which starch is extracted, abbreviated here as SCWC.

BCWC peels and sarcocarp are rich in phenolic compounds (Adrian et al., 1996;Luo et al., 2014; Nie et al., 2018), exhibiting strong antioxidant, antibacterial, and antitumor effects (Zhan et al., 2016) and inhibit acrylamide formation and are often used in traditional Chinese medicine to treat pharyngitis, laryngitis, enteritis, cough, hepatitis, and hypertension (Luo et al., 2014; Nie et al., 2018). BCWC is one of the most popular hydrophytic vegetables in China, owing to its unique flavor (Li et al., 2016). Fresh‐cut Chinese water chestnut has been used to preserve food products and beverages and is sold worldwide (Li et al., 2016; Luo et al., 2014).

The SCWC is rich in starch and is usually not consumed directly. Currently, it is used for vermicelli production during food processing (Tang et al., 2018), and leads to the production of large amounts of SCWC peels as waste. The peel of CWC is rich in bioactive components, which is benefit to human body. However, there are still few studies on the chemical constituents of CWC peels (Luo et al., 2014).

At present, there are a few reports regarding the types of flavonoids and phenolic acids present in the peels of BCWC and SCWC. Qualitative and quantitative transformation of nutrients and bioactive compounds is difficult. Liquid Chromatography–Tandem Mass Spectrometry (LC–MS/MS) is a rapid and highly sensitive method (Raclariu et al., 2017; Wang et al., 2019) that is used for the detection of metabolic products in comparison databases (Barraza‐Elenes et al., 2019; Wang, Li, et al., 2018; Wang, Zhang, et al., 2018; Frolov et al., 2013; Fang et al., 2003) and was used to identify and quantify metabolites present in BCWC and SCWC peels, especially flavonoids and organic acids and their derivatives.

In this study, we conducted an ultraperformance liquid chromatography/mass spectrometry (UPLC/MS)‐based metabolomics analysis to investigate the constituents of two CWC cultivars. The chemical compositions of the two cultivars of CWC were distinguished here, which is of great significance to the future utilization of CWC peels.

## 2 MATERIALS AND METHODS

### 2.1 Plant materials and samples

BCWC and SCWC were planted in the same field and under the same conditions at the same time, which is the typical size of the selected Chinese varieties (Fanglin Chinese water chestnut, harvested from Guangxi Province in southern China in November 2018), and identified by Prof. Yanghe Luo, Hezhou University (Hezhou, Guangxi, China). The peels of BCWC and SCWC were stored at −80°C until further analysis.

### 2.2 Sample preparation and extraction

Freeze‐dried BCWC and SCWC peels were crushed using a mixer mill (MM 400, Retsch, Germany) with a zirconia bead for 1.5 min at 30 Hz. Hundred milligrams of the powder was weighed and extracted overnight at 4°C using 1.0 ml of 70% aqueous methanol. The samples were then centrifuged at 10000 × g for 10 min, and the extracts were absorbed (CNWBOND Carbon‐GCB SPE Cartridge, 250 mg, 3 ml; ANPEL) and filtered (SCAA‐104, 0.22 μm pore size; ANPEL) for UPLC–MS analysis (Wang, Li, et al., 2018; Wang, Zhang, et al., 2018). Each experiment was repeated three times.

### 2.3 UPLC analysis

Samples were injected into the LC‐ESI‐MS/MS system (HPLC, Shim‐pack UFLC SHIMADZU CBM30A system, MS, Applied Biosystems QTRAP). The analytical conditions were as follows: HPLC: column, Waters ACQUITY UPLC HSS T3 C_18_ (1.8 μm, 2.1 mm × 100 mm); solvent system, mobile phase A: water (0.04% acetic acid); mobile phase B: acetonitrile (0.04% acetic acid), gradient program, 95:5 v/v at 0 min, 5:95 v/v at 11.0 min, 5:95 v/v at 12.0 min, 95:5 v/v at 12.1 min, 95: 5 v/v at 15.0 min; flow rate, 0.40 ml/min; temperature, 40°C; injection volume: 2 μl. The effluent was channeled into the ESI‐triple quadrupole‐linear ion trap (Q TRAP)‐MS.

### 2.4 ESI‐Q TRAP‐MS/MS analysis

Linear ion trap (LIT) and triple quadrupole (QQQ) scans were performed using a QQQ TRAP API Q TRAP LC/MS/MS System, equipped with an ESI Turbo Ion‐Spray interface, operating in the positive ion mode and controlled by Analyst 1.6.3 software (AB Sciex). Operation parameters of the ESI source were as follows: ion source, turbo spray; source temperature 500°C; ion spray voltage (IS) 5500 V; ion source gas I (GSI), gas II (GSII), and curtain gas (CUR) were set at 55, 60, and 25.0 psi, respectively; the collision gas (CAD) was high. Instrument tuning and mass calibration were performed with 10 and 100 μmol/L polypropylene glycol solutions in QQQ and LIT modes, respectively. QQQ scans were acquired in the multiple reaction monitoring (MRM) mode with the collision gas (nitrogen) set to 5 psi. DP and CE for individual MRM transitions were determined and further optimized (Chen et al., 2013). A specific set of MRM transitions was monitored for each period in accordance with metabolites eluted within said period.

### 2.5 Qualitative and quantitative analysis of metabolites

Qualitative analyses were conducted using the stepwise multiple‐ion monitoring‐enhanced product ions (MIM‐EPI) strategy and the MS2T data were analyzed by comparing the accurate precursor ion (Q1) and product ion (Q3) values, retention time (RT), and a self‐compiled database MWDB (Met Ware biological science and Technology Co., Ltd) and publicly available metabolite databases if the standards were unavailable (Chen et al., 2013; Wei et al., 2014). Isotope signals, repeated signals containing K^+^, Na^+^, and NH^+^, and repeated signals of further fractionated substances, can be determined through qualitative analysis of substances in accordance with their spectral data of K^+^, Na^+^, and NH^+^ obtained during fractionation. The results show that the fractionation of the fractionated substances is the same as that of the other substances in the fractionation (Pinnapat et al., 2018). Metabolomics data of BCWC and SCWC peels were processed using System Software Analyst (Version 1.6.3). The MRM model was quantitatively analyzed, and 2‐amino‐3‐(2‐chloro‐phenyl)‐propionic acid (1 ppm) as internal standard. Thus, information regarding the content and structure of 321 metabolites in BCWC and SCWC peels was obtained. Based on the metabolome database, 321 metabolites were qualitatively analyzed, and the potential structures of 88 flavonoids were putatively determined (Table 1).

**TABLE 1:** List of 87 flavonoid metabolites identified in *Eleocharis dulcis* (Burm. f.) Trin. ex Hensch

Metabolite name	Precursor ions (Q1) (Da)	Product ions (Q3) (Da)	Rt (min)	Molecular weight (Da)
Methyl Quercetin O‐hexoside	479	317.1	3.91	478
Selgin 5‐O‐hexoside	479.1	317.1	3.52	478.12
Tricin 5‐O‐acetylglucoside	353.1	331.1	4.69	534.11
Selgin O‐malonylhexoside	565	317	4.33	564
Tricin O‐rhamnosyl‐O‐malonylhexoside	725.2	331.1	4.5	724.2
Naringenin O‐malonylhexoside	521	273	4.5	520
Tricetin O‐malonylhexoside	551	303.1	4.56	550
Chrysin O‐malonylhexoside	503	255	5.24	502
Ayanin	345.2	177.2	6.33	344.2
Luteolin 6‐C‐glucoside	499.1	299.1	3.45	498.1
Apigenin O‐malonylhexoside	519	271	4.23	518
Luteolin 3′,7‐di‐O‐glucoside	611.1	449.3	3.32	610.1
Chrysoeriol O‐hexosyl‐O‐rutinoside	771.1	463.2	3.92	770.1
Chrysoeriol O‐hexosyl‐O‐pentoside	595.1	301.4	4.12	594.1
Chrysoeriol O‐hexosyl‐O‐hexoside	625.1	301.6	4.17	624.1
Chrysoeriol O‐malonylhexoside	549.1	301.4	4.55	348.1
6‐C‐hexosyl‐apigenin O‐hexosyl‐O‐hexoside	757.1	433.4	3.1	756.1
Eriodictiol C‐hexosyl‐O‐hexoside	613.1	451.1	3.09	612.1
Chrysoeriol 6‐C‐hexoside	463.1	313.1	3.04	462.1
6‐C‐hexosyl‐hesperetin O‐hexoside	627.1	465.2	3.41	626.1
C‐hexosyl‐luteolin O‐feruloylhexoside	787.2	463.1	3.64	786.2
Eriodictyol O‐malonylhexoside	537	289	4.41	536
8‐C‐hexosyl chrysoeriol O‐hexoside	625	463	3.46	624
Tricin 5‐O‐hexosyl‐O‐hexoside	655.2	333.1	3.5	654.2
Tricin 7‐O‐hexosyl‐O‐hexoside	655.2	333.2	3.37	654.2
Tricin di‐O‐hexoside	655.2	330.9	4.16	654.2
Tricin O‐malonylhexoside	579.1	331.1	4.56	578.1
Tricin 4’‐O‐(β‐guaiacylglyceryl) ether O‐hexoside	689.2	331	4.97	688.2
Tricin O‐glycerol	405.1	331.2	5.23	404.1
Tricin O‐hexosyl‐O‐syringin alcohol	659.3	331	4.92	658.3
Tricin 4’‐O‐(β‐guaiacylglyceryl) ether 7‐O‐hexoside	689.2	331.2	4.43	688.2
Tricin 4’‐O‐syringic acid	511.2	331.1	6.38	510.2
Tricin	329	314	5.73	530
Acetyl‐eriodictyol O‐hexoside	491.1	287.1	5.09	492.1
Chrysoeriol O‐hexosyl‐O‐malonylhexoside	709.1	547.1	2.42	710.1
Chrysoeriol 5‐O‐hexoside	461.1	299.1	3.87	462.1
Apigenin 7‐O‐glucoside (Cosmosiin)	431.1	269.1	4.21	432.1
Luteolin C‐hexoside	447.1	327.1	3.45	448.1
Tricin 5‐O‐hexoside	491.1	329.1	4	492.1
Tricin 4’‐O‐(syringyl alcohol) ether 5‐O‐hexoside	657.1	495.1	4.5	658.1
Tricin 4’‐O‐(syringyl alcohol) ether 7‐O‐hexoside	657.1	329.2	4.72	658.1
Tricin 4’‐O‐syringyl alcohol	495.1	329.1	5.84	496.1
Tricin 4’‐O‐β‐guaiacylglycerol	525.1	191.1	1.82	526.1
4’‐Hydroxy‐5,7‐dimethoxyflavanone	299.1	223.1	6.78	300.1
Luteolin	287.1	153	5	286.1
Quercetin 3‐O‐rutinoside	609.2	301	3.7	610.15
Quercetin	301	151	5.12	302.4
Chrysoeriol	299.1	284	5.76	300.06
Naringenin 7‐O‐glucoside	433.1	271	4.22	434.12
Isovitexin	431.1	311	3.79	432.11
Naringenin	271.1	151	5.59	272.07
Apigenin	271.1	153	5.63	270.05
Phloretin	273.1	167	5.56	274.08
Kaempferol 7‐O‐rhamnoside	433.1	287	4.94	432.11
Acacetin	285.1	270	7.06	284.07
Genistein (4′,5,7‐Trihydroxyisoflavone)	271.1	215	5.52	270.05
7‐O‐Methyleriodictyol	301.1	165	6.28	302.08
Sakuranetin	287.1	167	6.96	286.08
Isovitexin 7‐O‐glucoside	595.2	415	3.36	594.16
Hesperetin	301.1	164	5.75	302.08
Luteolin 7‐O‐glucoside	447.3	285.2	3.87	448.1
Dihydromyricetin	319.1	301	3.52	320.05
Naringenin chalcone	271.1	151	5.57	272.07
Isosakuranetin‐7‐neohesperidoside	593	285	5.09	594.2
Quercetin 4’‐O‐glucoside	465	303	3.86	464.1
Calycosin	283	268	5	284.07
Glycitein	283	268	5.13	284.07
Kaempferol 3‐O‐galactoside	447	285	3.86	448.1
3,7‐Di‐O‐methylquercetin	329	314	6.63	330.07
Prunetin	283	268	6.97	284.07
Rhamnetin (7‐O‐methxyl quercetin)	315	165	6.43	316.06
Laricitrin	333	153	5.15	332.05
Homoeriodictyol	301.1	151	5.75	302.28
Isosakuranetin (4′‐Methylnaringenin)	285.1	164	6.98	286.08
Butin	271.1	151	5.59	272.07
Morin	301	151	5.11	302.04
4,2′,4′,6’‐Tetrahydroxychalcone	271.1	151	5.66	272.07
Vestitol	271.1	134.8	6.27	272.1
Hyperoside	465.1	303	3.73	464.1
Tiliroside	593.1	285	4.92	594.14
Orientin	449.1	299.2	3.46	448.1
5,7‐Dihydroxychromone	177	88.9	4.47	178.03
Herbacetin	303	121.3	5.13	302.04
Pedalitin	315.1	300	4.72	316.06
5,7‐Dihydroxy‐3′,4′,5′‐trimethoxyflavone	343.1	313	6.81	344.09
Persicogenin	315.1	178	7.15	316.09
Tectorigenin	299.1	211	5.72	300.06

### 2.6 Multivariate and cluster analysis of BCWC and SCWC peels

**FIGURE 1:**
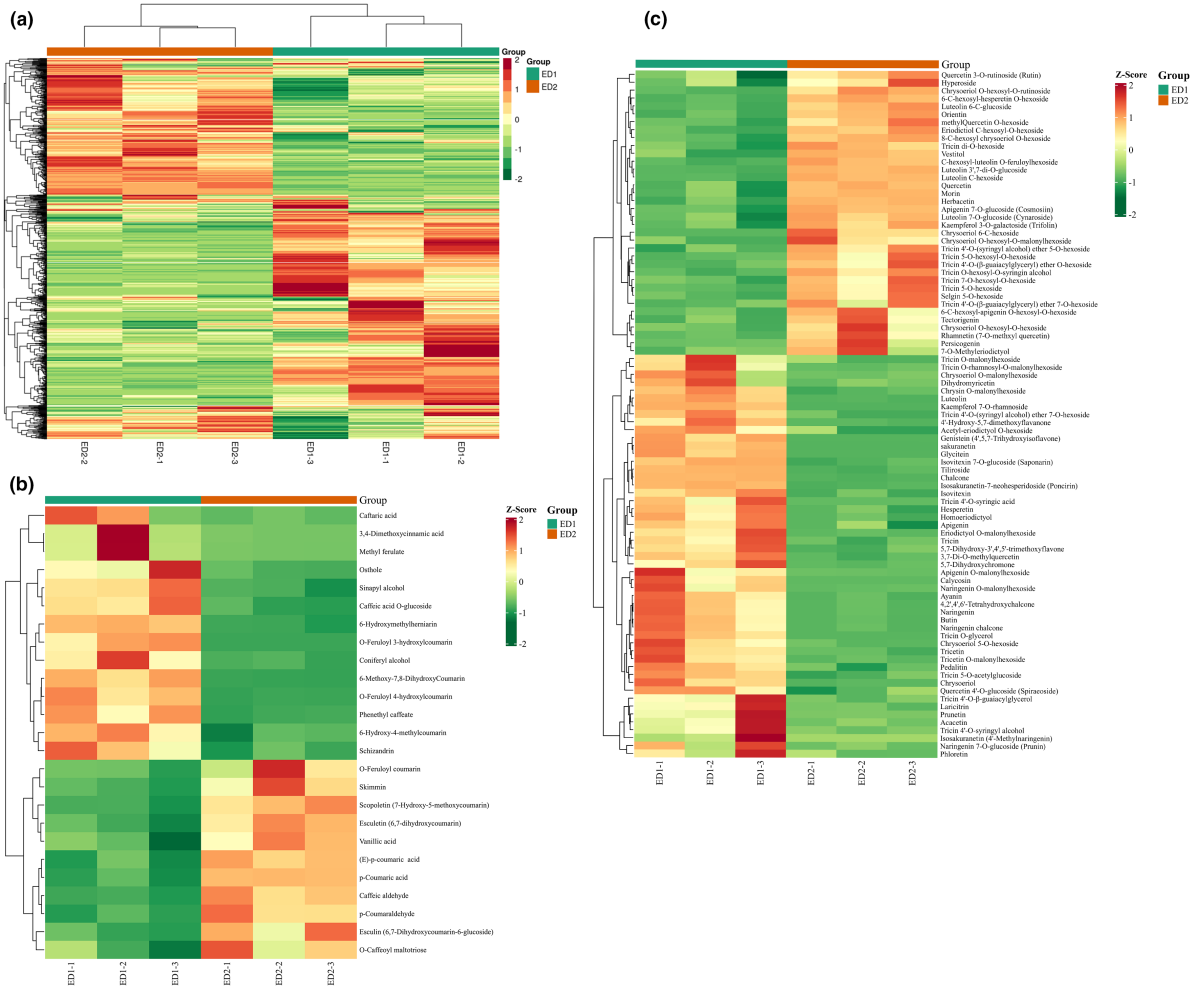
Heat map visualization all of metabolomics (a), phenylpropanoids (b), and flavonoids (c). The content of each metabolite was normalized to complete linkage hierarchical clustering. Each example can be visualized in a single column and each metabolite is represented by a single row. Red indicates high abundance, while relatively low metabolites are shown in green (color key scale presented to the right of the heat map)

All 321 metabolomics were analyzed. To eliminate the effect of concentration on pattern recognition, the logarithm (log10) of the peak area matrix of BCWC and SCWC metabolites was determined, which was followed by Poisson normalization (Xia et al., 2015). Thereafter, cluster analysis of the metabolite profiles of BCWC and SCWC peels was conducted using LC–MS/MS analysis. Results obtained using BCWC and SCWC peels were dichotomized as follows (Figure 1a **)**: values for BCWC and SCWC peels were segregated in the PCA score plot of sesame metabolites. Furthermore, they were clearly divided into two classes on the heat map, indicating significant differences in levels of secondary metabolites in BCWC and SCWC peels.

## 3 RESULTS AND DISCUSSIONS

### 3.1 Metabolic profiling

Analysis of the chemical constituents of the two CWC species revealed that they are rich in flavonoids. To confirm differences in total contents and composition, a new LC–MS‐based method that is widely used for metabolomics was employed herein (Dong et al., 2015; Wei et al., 2014). Consequently, 321 significant metabolites (87 flavonoids, 43 organic acids, and derivatives, 40 lipids, 31 amino acid and derivatives, 25 phenylpropanoids, 22 nucleotide and derivates, 18 alkaloids, 13 terpenes, 11 alcohols, 7 phenolamides, 6 carbohydrates, 4 vitamins and derivatives, 3 indole derivatives, 3 polyphenols, 3 anthocyanins, 1 quinone, and 3 others) were identified/annotated (Supplementary S1). To observe differences in metabolites between BCWC and SCWC, differential metabolite screening was performed for all metabolites identified/annotated in accordance with the fold‐change and the variables considered important for the projection (VIP) scores. The criteria for significance of differences included a fold change value of *≥*2 or *≤*0.5 and a VIP value of *≥*1. These metabolites are shown in a heat map, indicating significant differences in the metabolite levels between BCWC and SCWC (Figure 1a).

**FIGURE 2:**
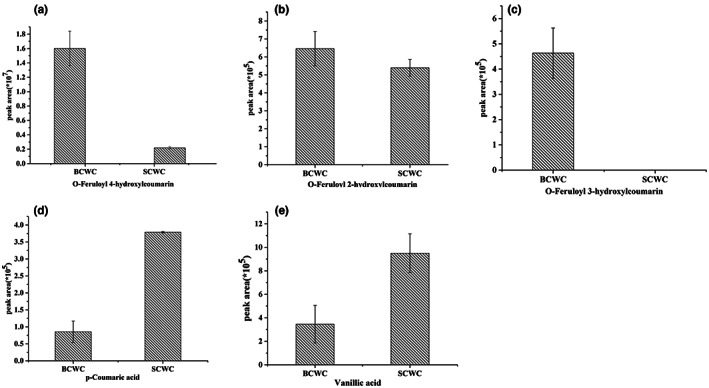
Phenylpropanoids content in BCWC and SCWC

We detected 25 phenylpropanoids (Figure 1b), including *O*‐feruloyl 4‐hydroxycoumarin, caffeic aldehyde, *O*‐feruloyl coumarin, caftaric acid, coniferyl alcohol, and osthole, which displayed better antimicrobial activity (Nie et al., 2016). Herein, three isomers of *O*‐feruloyl‐4‐hydroxycoumarin, *O*‐feruloyl‐2‐hydroxycoumarinwere, and *O*‐feruloyl‐3‐hydroxycoumarin markedly higher in BCWC than in SCWC (Figure 2a–c) while p‐coumaric acid and vanillic acid levels were markedly higher in SCWC than in BCWC (Figure 2d,e).

**FIGURE 3:**
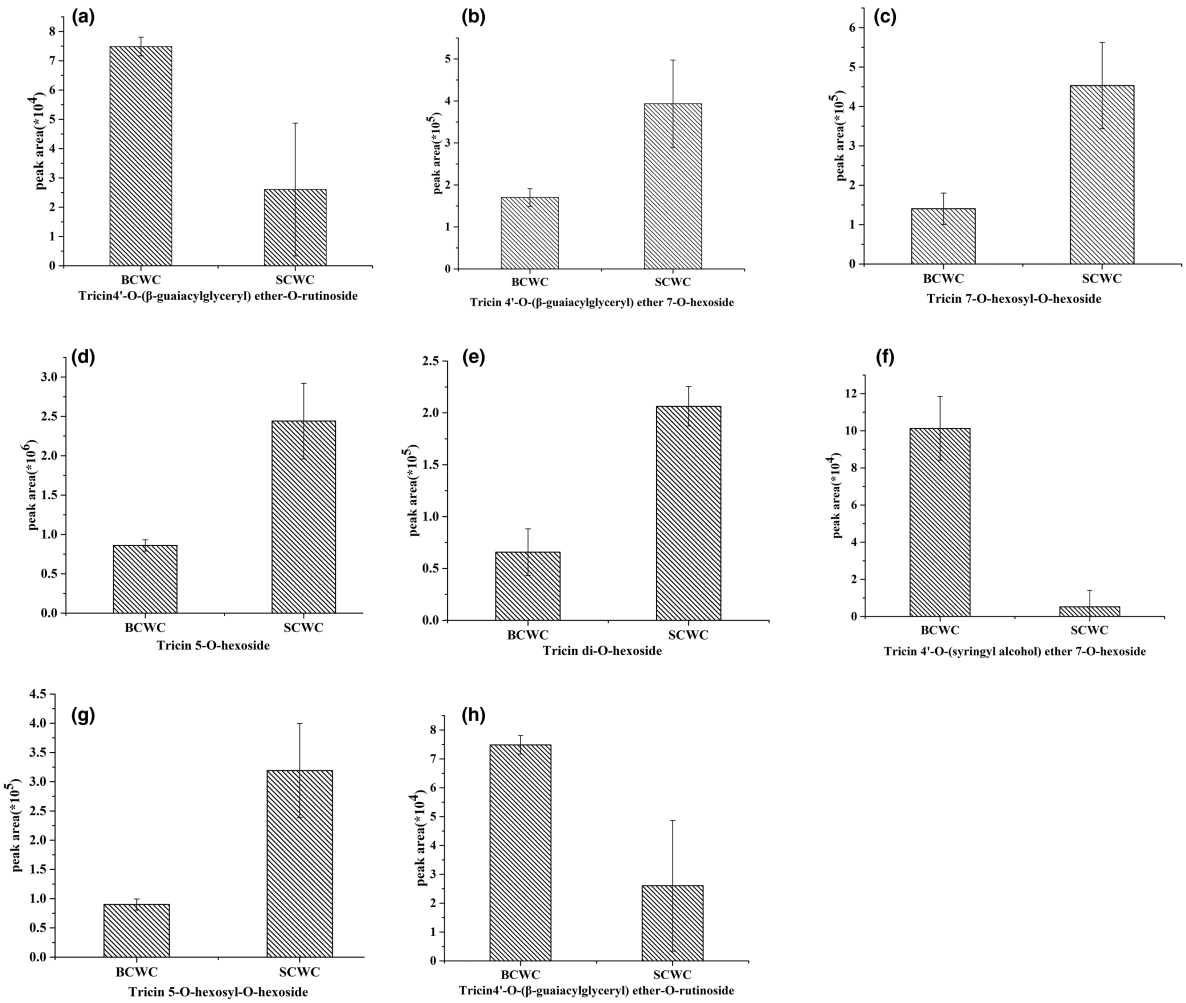
Flavonoids content in BCWC and SCWC

We identified 87 flavonoids in total, including 12 flavonols, 12 flavanones, 60 flavones, and 3 anthocyanins (Figure 1c). Glycosides mostly displayed 3‐*O*, 5‐*O*, and 7‐*O* linkages. Both BCWC and SCWC peels were rich in tricin, which primarily presented *O*‐hexosides, 5‐*O*‐hexosides, and 7‐*O*‐hexosides (Figure 3a‐h **)**. Furthermore, high levels of chrysoeriol were observed in both BCWC and SCWC peels, and it exhibited *O*‐hexoside or *O*‐rutinoside modifications. Unlike rice, no C‐glycosides were detected in BCWC and SCWC peels (Dong et al., 2015). It was difficult to identify individual anthocyanins because of complex glycosylation patterns (Li et al., 2012). We only identified three anthocyanins: malvidin 3‐*O*‐glucoside, peonidin‐*O*‐hexoside, and peonidin‐3‐*O*‐glucoside chloride, as reported previously (Duan et al., 2015; Martín et al., 2015; Zhang et al., 2015).

### 3.2 Differential metabolite analysis based on PCA

**FIGURE 4:**
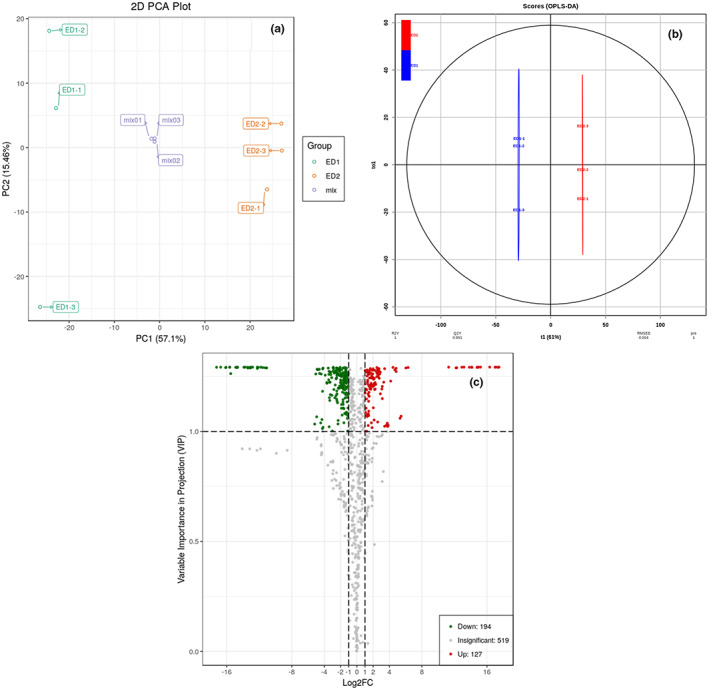
PCA and OPLS‐DA of the relative differences in metabolites in different CWC cultivars. a: Score plots for principle components 1 and 2 showed high cohesion within groups and good separation between two CWC cultivars from the BCWC and SCWC. The sampling groups are color coded as follows: Green = ED1; orange = ED2; and mustard = QC samples. b: 0PLS‐DA model plots and loading plots for the BCWC (ED1) and SCWC (ED2). c: The volcano plot shows the differential metabolite expression levels between BCWC and SCWC. Green dots represent downregulated differentially expressed metabolites; red spots represent upregulated differentially expressed metabolites, and gray represents insignificantly expressed metabolites

Principal component analysis (PCA) is often used to study the internal structure of multiple variables using a few principal components or to derive a few principal components from the original variable so that they can retain as much information about the original variable as possible. Moreover, these components/variables are not related to each other, and usually, a mathematical formula is used to represent the original indicators as a linear combination or a new comprehensive index. In the PCA plot (Figure 4a), PC1 and PC2 were 57.1% and 15.46%, respectively. ED_1_ and ED_2_ showed a clear distinction between the samples, indicating that there is a large difference in metabolites between BCWC and SCWC.

### 3.3 Differential metabolite analysis via partial least squares‐discriminant analysis (OPLS‐DA)

OPLS‐DA is more sensitive to variables with low correlations (Thévenot et al., 2015). The constituents of SCWC and BCWC were compared to identify the metabolites responsible for the observed differences. OPLS‐DA models were used to carry out pairwise comparisons of these metabolites. High predictability (Q^2^) and strong goodness of fit (R^2^X, R^2^Y) of the OPLS‐DA models were observed on comparing BCWC and SCWC (Q^2^ = 0.963, R^2^X = 0.599, R^2^Y = 0.995, Figure 4b). A Q^2^ value greater than 0.9 indicated an excellent model, while that greater than 0.5 indicated an effective model. The OPLS‐DA models for BCWC were distinct from those for SCWC.

It can be seen from the volcanic map (Figure 4c). Compared with SCWC (ED2), 127 chemical components of BCWC (ED1) are upregulated and 194 chemical components are downregulated, indicating that BCWC (ED1) has more abundant active components.

### 3.4 Differential metabolic pathways in BCWC and SCWC

Differential metabolites between BCWC and SCWC were mapped to the KEGG database to obtain detailed information regarding metabolic pathways they may participate in (Figure S2). The KEGG database facilitates studies on gene expression and function and metabolite content as a complete network. As a primary public database for pathways, KEGG provides information regarding integrated metabolic pathways (pathway), including the metabolism of carbohydrates, nucleosides, and amino acids and biodegradation of organic matter, thus indicating all potential metabolic pathways (Supplementary S3). Moreover, enzymes catalyzing each step of the reaction are comprehensively annotated in this database, including amino acid sequences and links to the PDB library, which makes it a powerful tool for metabolomic analysis and studies on metabolic networks in vivo.

It can be seen from Supplementary S3 that there are nine metabolic pathways in BCWC and SCWC that are focused on KEGG, including metabolic pathways (ko: 01100; 51.30%), biosynthesis of secondary metabolites (ko: 01110; 38.20%), flavone and flavonol biosynthesis (ko: 00944; 19.30%), flavonoid biosynthesis (ko: 00941; 18.10%) et al. Among them, the biosynthetic pathway of flavonoids and phenylpropanoid compounds has been relatively clear (Li et al., 2016). The compounds of flavonol, flavone, flavanone, and isoflavone are come from flavonoid biosynthesis, flavone and flavonol biosynthesis, biosynthesis of secondary metabolites, and biosynthesis of phenylpropanoids, while the compounds of phenylpropanoids are come from metabolic pathways, biosynthesis of secondary metabolites, and phenylpropanoid biosynthesis.

## 4 CONCLUSION

In the present study, the chemical profiles of BCWC and SCWC were analyzed using the widely targeted metabolomics method and metabolite compounds were identified and classified.

Marked differences were observed in the metabolites of SCWC and BCWC. In total, 321 differential metabolites were identified, comprising flavonoids, phenylpropanoids, organic acids, and derivatives. In particular, 15 alkaloids and 13 terpenes were identified, for the first time, in *Eleocharis dulcis* (Burm. f.) Trin. ex Hensch.

CWC is among the most widespread and economically important vegetables, with a wide array of uses in the food and medicine industries. To the best of our knowledge, the difference in chemical profiles between different cultivars may inform different functions. Our work focused on the different flavonoids and phenylpropanoids contained in the two different CWC cultivars. In total, 87 flavonoids were identified with different levels in BCWC and SCWC. We also identified 25 phenylpropanoids, of which higher levels were found in SCWC than in BCWC. Feruloyl and dihydroxycoumarin were the predominant phenylpropanoids in SCWC. Our results help further the current understanding of metabolic mechanisms accounting for the differences in different cultivars of *Eleocharis dulcis* (Burm. f.) Trin. ex Hensch.

## FUNDING INFORMATION

The National Key R&D Program of China, Grant/Award Number: 2018YFD0901003; Natural Science Foundation of Guangxi, China, Grant/Award Number: 2020GXNSFBA297083; The Project to Improve the Basic Research Ability of Young and Middle‐Aged Teachers in Guangxi, China, Grant/Award Number: 2021KY0704; School Level Project of Hezhou University, China, Grant/Award Number: 209200214.

## CONFLICT OF INTEREST

The authors declare no competing financial interest.

## ETHICS STATEMENT

This research does not include any human or creature testing.

## Supporting information


Supinfo S1

Figure S2

Supinfo S3

## Data Availability

The data will be available from the authors upon request.
