# Supplementary figures and images for: Identification of compounds from chufa (Eleocharis dulcis) peels by widely targeted metabolomics
Source: Food Sci Nutr. 2022 Oct 3;11(1):545–54. doi: 10.1002/fsn3.3085 (PMC9834879; doi:10.1002/fsn3.3085)

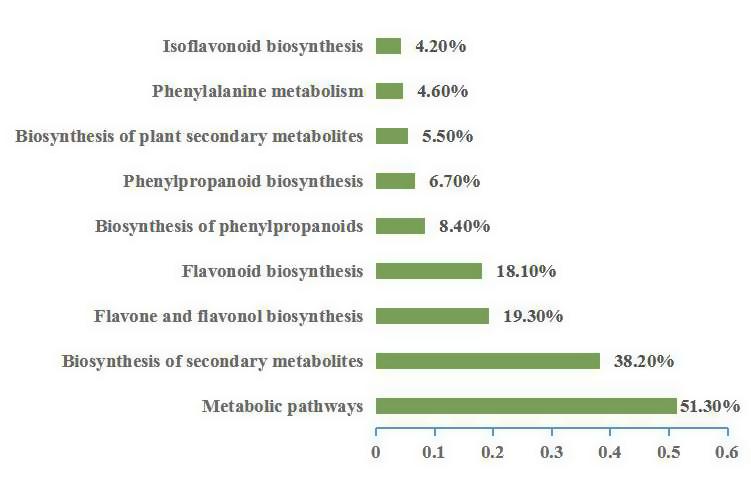

Supplement: Supplementary file 2 — Figure S2 [file FSN3-11-545-s003.jpg]
